# Efficacy and Safety of Pazopanib in the Treatment of Thyroid Cancer: A Systematic Review

**DOI:** 10.3390/biomedicines12122820

**Published:** 2024-12-12

**Authors:** Alexandra Laura Mederle, Loredana Gabriela Stana, Adrian Cosmin Ilie, Claudia Borza, Caius Glad Streian, Daciana Nistor, Teodor Cerbulescu, Biliana Belovan, Ana Lascu

**Affiliations:** 1 Surgery Clinic, County Emergency Hospital “Pius Brinzeu”, 300723 Timisoara, Romania; alexandra.mederle@umft.ro; 2 Faculty of Medicine, “Victor Babes” University of Medicine and Pharmacy, 300041 Timisoara, Romania; 3 Department I, Discipline of Anatomy and Embriology, “Victor Babes” University of Medicine and Pharmacy, 300041 Timisoara, Romania; loredana.stana@umft.ro; 4 Department III Functional Sciences, Division of Public Health and Management, “Victor Babes” University of Medicine and Pharmacy, 300041 Timisoara, Romania; ilie.adrian@umft.ro; 5 Department of Functional Sciences, Discipline of Physiopathology, Faculty of Medicine, “Victor Babes” University of Medicine and Pharmacy, 300041 Timisoara, Romania; borza.claudia@umft.ro (C.B.); lascu.ana@umft.ro (A.L.); 6 Centre for Translational Research and Systems Medicine, “Victor Babes” University of Medicine and Pharmacy, 300041 Timisoara, Romania; 7 Centre of Cognitive Research in Pathological Neuro-Psychiatry (NEUROPSY-COG), “Victor Babes” University of Medicine and Pharmacy, 300041 Timisoara, Romania; 8 Department VI Cardiology-Cardiovascular Surgery, “Victor Babes” University of Medicine and Pharmacy, 300041 Timisoara, Romania; streian.caius@umft.ro; 9 Advanced Research Center of the Institute for Cardiovascular Diseases, “Victor Babes” University of Medicine and Pharmacy, 300041 Timisoara, Romania; 10 Institute for Cardiovascular Diseases of Timisoara, Clinic for Cardiovascular Surgery, 300310 Timisoara, Romania; 11 Department of Functional Sciences, Physiology and Biotechnologies (CIFBIOTEH), “Victor Babes” University of Medicine and Pharmacy, 300041 Timisoara, Romania; 12 Centre for Gene and Cellular Therapies in Cancer, 3000723 Timisoara, Romania; 13 Department III—Microscopic Morphology, Discipline of Cellular and Molecular Biology, “Victor Babes” University of Medicine and Pharmacy, Eftimie Murgu Square 2, 300041 Timisoara, Romania; cerbulescuteodor28@gmail.com; 14 Doctoral School, “Victor Babes” University of Medicine and Pharmacy, Eftimie Murgu Square 2, 300041 Timisoara, Romania; belovan.biliana@umft.ro

**Keywords:** pazopanib, thyroid cancer, medullary thyroid carcinoma, differentiated thyroid cancer, anaplastic thyroid carcinoma, systematic review, efficacy, safety

## Abstract

Pazopanib, a multi-targeted tyrosine kinase inhibitor, has been explored for its efficacy in treating various subtypes of thyroid cancer, including differentiated thyroid carcinoma (DTC), medullary thyroid carcinoma (MTC), and anaplastic thyroid carcinoma (ATC). This systematic review assesses the efficacy and safety of pazopanib, focusing on the progression-free survival (PFS), overall survival (OS), and response rates and adverse events. A comprehensive search was conducted in databases including PubMed, Scopus, and Web of Science up to October 2024 to identify randomized controlled trials and phase II clinical trials that investigated the use of pazopanib in thyroid cancer. The PRISMA guidelines were followed for data extraction and quality assessment. The review included six studies encompassing 289 patients, presenting a comprehensive overview of pazopanib’s application across different thyroid cancer subtypes. The studies reported median PFS rates ranging from 2.1 to 11.7 months and median OS rates ranging from 5.7 months to not reached. The partial response rates varied from 5% to 49%. Adverse events were common, with hypertension occurring in up to 71.7% of patients, and fatigue and diarrhea were also frequently reported. Grade 3–5 adverse events led to treatment discontinuations in up to 14% of patients. Pazopanib shows variable efficacy across thyroid cancer types, offering significant benefits in MTC and refractory DTC in terms of PFS and OS but limited impact in ATC. The adverse event profile necessitates careful management, particularly regarding hypertension and other treatment-related toxicities. Further studies are required to refine the therapeutic protocols for pazopanib, exploring combination therapies that may enhance its efficacy and reduce the adverse outcomes.

## 1. Introduction

Thyroid cancer is the most prevalent endocrine malignancy, with an increasing incidence rate worldwide over the past few decades [1,2]. It encompasses a diverse group of tumors derived from thyroid follicular epithelial cells or parafollicular C cells, leading to different histological subtypes such as differentiated thyroid carcinoma (DTC), medullary thyroid carcinoma (MTC), and anaplastic thyroid carcinoma (ATC) [3,4]. DTC, which includes papillary and follicular thyroid cancers, accounts for the majority of cases and generally has an excellent prognosis when diagnosed early and treated appropriately [5,6]. However, a subset of patients develops aggressive disease that is refractory to conventional therapies. MTC, arising from parafollicular C cells, represents approximately 2–4% of thyroid cancers and often presents with more aggressive behavior [7]. ATC is a rare but highly aggressive form, accounting for less than 2% of thyroid cancers but contributing to a disproportionate number of thyroid-cancer-related deaths due to its rapid progression and poor response to treatment [8].

The development of thyroid cancer is influenced by a convergence of genetic, environmental, and hormonal factors. Central to the pathogenesis of thyroid cancer are genetic mutations and alterations in key signaling pathways, such as the MAPK/ERK and PI3K/AKT pathways, which are crucial for cell growth and survival [9]. Mutations in genes such as BRAF, RET/PTC, and RAS lead to uncontrolled cell proliferation and tumor growth [10]. Environmental factors, including radiation exposure, particularly during childhood, significantly elevate the risk [10]. Additionally, hormonal influences, with a higher incidence rate in females, suggest a role for estrogen in modulating tumor growth and thyroid gland physiology. The management of advanced thyroid cancer poses significant clinical challenges. While surgery and radioactive iodine (RAI) therapy are effective for early-stage DTC, patients with RAI-refractory DTC have limited treatment options and a poorer prognosis [11]. Similarly, MTC does not take up RAI, and traditional chemotherapy has shown limited efficacy [12]. ATC, with its aggressive nature, often presents at an advanced stage and is resistant to most conventional therapies [13]. The survival rates for patients with advanced MTC and ATC remain low, highlighting the urgent need for novel therapeutic approaches that can improve outcomes in these patient populations [14]. Recent advances in understanding the molecular pathways involved in thyroid carcinogenesis have opened new avenues for targeted therapies.

Angiogenesis plays a crucial role in the growth and metastasis of thyroid cancers [15]. The overexpression of vascular endothelial growth factor (VEGF) and its receptors has been observed in various thyroid cancer subtypes, promoting neovascularization and tumor progression [15]. Targeting angiogenesis through the inhibition of VEGF receptors has emerged as a promising strategy in cancer therapy. Additionally, mutations in tyrosine kinase receptors, such as RET mutations in MTC, contribute to tumor proliferation and survival [16]. Therefore, multi-targeted tyrosine kinase inhibitors (TKIs) that can inhibit angiogenesis and oncogenic signaling pathways offer potential therapeutic benefits in advanced thyroid cancers.

Pazopanib, with the IUPAC name 5-[[4-[(2,3-dimethyl-2H-indazol-6-yl)methylamino]pyrimidin-2-yl]amino]-2-methylbenzenesulfonamide, features a complex chemical structure that includes a pyrimidine ring linked to a dimethyl-indazole and a toluenesulfonamide group. Pazopanib is an oral, multi-targeted TKI that inhibits VEGFR-1, -2, and -3; platelet-derived growth factor receptors (PDGFR)-α and -β; fibroblast growth factor receptors (FGFR); and c-Kit [11]. It is approved for the treatment of advanced renal cell carcinoma and soft tissue sarcoma [17]. By blocking these receptors, pazopanib can inhibit angiogenesis and tumor cell proliferation [18]. The rationale for investigating pazopanib in thyroid cancer is based on its ability to target pathways involved in tumor growth and angiogenesis that are relevant to thyroid carcinogenesis. Preclinical studies have shown that pazopanib can inhibit the proliferation of thyroid cancer cell lines, providing a basis for clinical investigation [19].

This systematic review aims to evaluate the efficacy and safety of pazopanib in the treatment of thyroid cancer, including DTC, MTC, and ATC. By synthesizing data from clinical studies, we seek to provide a comprehensive understanding of pazopanib’s therapeutic potential in these malignancies. The outcomes of interest include the progression-free survival, overall survival, and objective response rates and adverse events. The findings of this review will inform clinical practice and guide future research directions in the management of advanced thyroid cancers.

## 2. Materials and Methods

### 2.1. Eligibility Criteria

This systematic review included studies that met the following inclusion criteria: (1) clinical studies involving patients diagnosed with thyroid cancer, specifically differentiated thyroid carcinoma (DTC), medullary thyroid carcinoma (MTC), or anaplastic thyroid carcinoma (ATC), treated with pazopanib as monotherapy or in combination with other therapies; (2) studies reporting on clinical outcomes such as the progression-free survival (PFS), overall survival (OS), objective response rate (ORR), disease control rate (DCR), biochemical response, and safety profiles, including adverse events (AEs); (3) randomized controlled trials (RCTs), phase II clinical trials, and prospective cohort studies to ensure high-quality evidence; (4) studies published in English to ensure comprehensive understanding and accurate data extraction. The exclusion criteria were: (1) studies not involving human subjects, such as preclinical laboratory studies or animal models; (2) case reports, case series, reviews, commentaries, and editorials that did not present original data; (3) studies lacking sufficient data on clinical outcomes or safety profiles specific to pazopanib treatment; (4) studies where pazopanib was not the primary intervention or its effects could not be isolated from other treatments; (5) non-peer-reviewed articles or grey literature to maintain the quality and reliability of the data.

### 2.2. Information Sources

A comprehensive literature search was conducted using three electronic databases: PubMed, Scopus, and Web of Science. The search was performed up to October 2024 to include the most recent and relevant studies on pazopanib in thyroid cancer. These databases were chosen due to their extensive coverage of biomedical and clinical research publications. Additionally, the reference lists of relevant articles were manually searched to identify any further studies that met the inclusion criteria. The systematic review was conducted following the Preferred Reporting Items for Systematic Reviews and Meta-Analyses (PRISMA) guidelines to ensure methodological rigor and transparency. The study protocol was registered in the Open Science Framework with the registration code osf.io/958pt.

### 2.3. Search Strategy

The search strategy was designed to capture all relevant studies on pazopanib in thyroid cancer. The key search terms included combinations of Medical Subject Headings (MeSH) and keywords such as “pazopanib”, “thyroid cancer”, “differentiated thyroid cancer”, “medullary thyroid carcinoma”, “anaplastic thyroid carcinoma”, “radioactive iodine-refractory”, “clinical trial”, “progression-free survival”, “overall survival”, “response rate”, and “adverse events”. Boolean operators (AND, OR) were used to combine search terms effectively. For example, the search string in PubMed was: (“pazopanib”) AND (“thyroid cancer” OR “differentiated thyroid cancer” OR “medullary thyroid carcinoma” OR “anaplastic thyroid carcinoma”) AND (“clinical trial” OR “progression-free survival” OR “response rate”). The search was limited to studies published in English and involving human subjects.

### 2.4. Selection Process

All identified records were imported into reference management software and duplicates were removed. Two independent reviewers screened the titles and abstracts for relevance based on the inclusion and exclusion criteria. Full-text articles of potentially relevant studies were retrieved and assessed independently by the reviewers. Any discrepancies during study selection were resolved through discussion or consultation with a third reviewer. The selection process was documented using the PRISMA flow diagram to ensure transparency and reproducibility.

### 2.5. Data Collection and Quality Assessment

The data extraction was performed independently by two reviewers using a standardized data extraction form. The extracted data included study characteristics (author, year, country, study design), patient demographics (sample size, age, sex), disease characteristics (type of thyroid cancer, stage, prior treatments), intervention details (pazopanib dosage, treatment duration), outcomes (PFS, OS, ORR, DCR, biochemical responses), and safety profiles (AEs, grade of toxicity). Where available, numerical data such as hazard ratios, median survival times, and response rates were recorded. Any disagreements in data extraction were resolved through discussion. The quality of the included studies was assessed using appropriate tools such as the Newcastle–Ottawa Scale for observational studies and the Cochrane Risk of Bias tool for randomized trials [20]. The extracted data were synthesized narratively, and where possible quantitatively, in tables to facilitate the analysis.

## 3. Results

### 3.1. Characteristics of Included Studies

**Figure 1:**
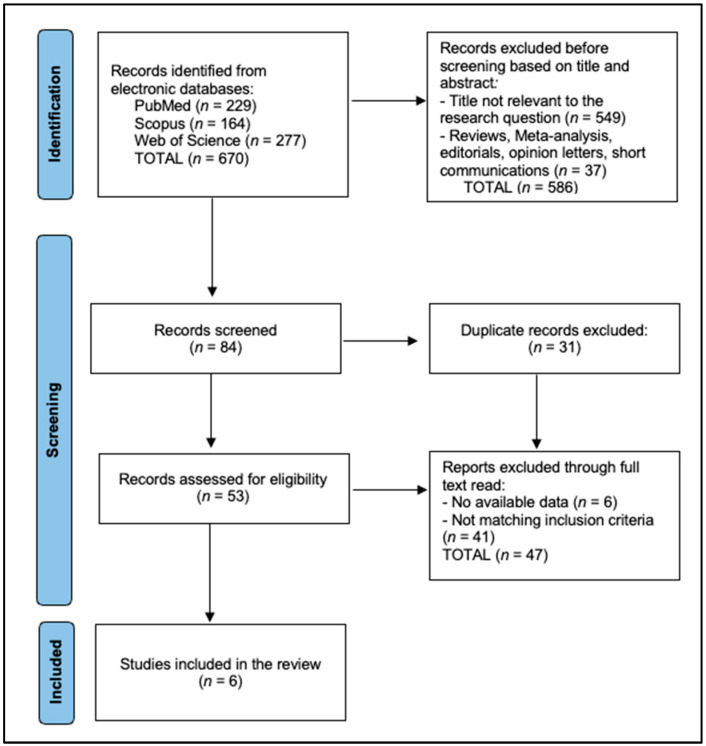
PRISMA flow diagram.

The final analysis included 6 studies [11,21,22,23,24,25], as presented in Figure 1. Collectively, these studies encompassed a total of 289 patients, illustrating a variety of designs and methodologies and showcasing the broad application of pazopanib across different thyroid cancer subtypes. Table 1 encapsulates the characteristics of six studies included in this systematic review on the effectiveness of pazopanib in thyroid cancers. Study 1 by Bible et al. [11], conducted in 2010 in the USA, was a multicenter phase II trial involving 37 patients with medullary thyroid carcinoma, assessed as high quality due to its multicenter approach and patient volume. Study 2 by Chow et al. [21], a phase I trial from 2017, also in the USA, included just 6 patients with well-differentiated thyroid carcinoma, slightly refractory to radioiodine, and was noted as being of moderate quality owing to its small sample size and preliminary phase nature. Study 3 by Sherman et al. [22] described a high-quality, randomized phase II trial from 2023 across 34 USA institutions, enrolling 89 patients with anaplastic thyroid carcinoma, 71 of whom were eligible for analysis. Study 4 by de la Fouchardière et al. [23] from France in 2021 involved 168 patients in a randomized phase II trial (PAZOTHYR), with 100 randomized, noted for its rigorous randomized design enhancing its quality. Study 5 by Bible et al. [24], another high-quality, multicenter phase II trial conducted in the USA in 2012, involved 15 patients with progressive medullary thyroid carcinoma. Lastly, Study 6 by Bible et al. [25] was an international phase II trial from 2020.

### 3.2. Patient Demographics and Baseline Characteristics

Table 2 provides a detailed view of the patient demographics and baseline characteristics across six studies in a systematic review investigating pazopanib in various thyroid cancer settings. In the study by Bible et al. [11], the 37 patients with RAI-refractory differentiated thyroid carcinoma (DTC) had a median age of 59 years, with a slight female predominance (20 females, 17 males). These patients had undergone surgery and radioactive iodine (RAI) therapy, with some having prior molecular kinase inhibitors (MKIs) exposure. Chow et al. [21] analyzed a small cohort of 6 patients with well-differentiated thyroid carcinoma (WDTC), presenting a mean age of 57.5 years and a male predominance (4 males, 2 females), all of whom had surgery, RAI, and some exposure to tyrosine kinase inhibitors (TKIs).

Sherman et al. [22] reported on 89 patients (71 eligible) with anaplastic thyroid carcinoma (ATC), showing a median age of 65 years and a balanced gender distribution (34 males, 37 females), who had undergone surgery but no prior systemic therapy for ATC. De la Fouchardière et al. [23] focused on 168 patients (100 randomized) with RAI-refractory DTC, where the median age was 67 years and showing a balanced gender ratio (81 males, 87 females), all treated with surgery, RAI, and some prior MKIs.

Bible et al. [24], in their study, included 15 patients with ATC, with a median age of 66 years and a male predominance (10 males, 5 females), who had undergone surgery, radiation, and prior systemic therapies. Lastly, Bible et al. [25] evaluated 60 patients with RAI-refractory DTC, where the median age was 60 years with a near equal gender distribution (33 males, 27 females), all having undergone surgery, RAI, and various systemic therapies.

### 3.3. Treatment Details and Efficacy Outcomes

Table 3 details the treatment protocols and efficacy outcomes of pazopanib across six studies included in a systematic review focusing on different thyroid cancer subtypes. In Bible et al.’s study [11], the patients received pazopanib at a dosage of 800 mg daily until progression or intolerability was noted. The treatment resulted in a median progression-free survival (PFS) period of 11.7 months, with a high partial response rate of 49%, while the median overall survival (OS) period was not reached, indicating strong activity in this patient group. Chow et al. [21] treated their cohort of 6 patients with pazopanib at 800 mg daily for 12 weeks, combined with escalating doses of radioactive iodine. The median PFS period was 6.7 months, with 80% of patients achieving stable disease, although the treatment showed no significant impact in terms of improving the iodine uptake in refractory well-differentiated thyroid carcinoma (WDTC) patients.

Sherman et al. [22] administered pazopanib in patients with anaplastic thyroid carcinoma (ATC) at 400 mg daily before and 300 mg during intensity-modulated radiation therapy (IMRT), alongside paclitaxel. The median OS period for patients in the pazopanib arm was 5.7 months, showing no significant survival advantage over the placebo arm, which suggests the limited efficacy of pazopanib when combined with other therapies in this aggressive cancer type. De la Fouchardière et al. [23] used a continuous or intermittent dosing regimen of 800 mg daily of pazopanib in their study. The median time to treatment failure (TTF) results were not significantly different between dosing schedules, with an overall response rate (ORR) of 5% after randomization, indicating minimal benefit from intermittent dosing.

Bible et al. [24] provided patients with pazopanib at 800 mg daily until progression or intolerability. The treatment yielded a median PFS period of only 2.1 months, with no confirmed responses according to the RECIST criteria, reflecting minimal clinical activity in this specific setting. Lastly, the other study by Bible et al. [25] treated their patients with pazopanib at 800 mg daily until progression or intolerability was observed. This regimen resulted in a median PFS of 11.4 months and a partial response rate of 36.7%, with a median OS of 2.6 years, showing some efficacy in managing refractory differentiated thyroid carcinoma.

### 3.4. Safety Profiles and Adverse Events

Table 4 compiles the safety profiles and adverse events reported across six studies involving the use of pazopanib in thyroid cancer treatment. In Bible et al.’s study [11], the common adverse events included hypertension (47%), diarrhea (49%), and fatigue (51%), with grade 3–4 adverse events reported in 46% of patients and one instance of a grade 5 pulmonary hemorrhage. Treatment discontinuation due to adverse effects occurred in 14% of patients. Chow et al. [21] noted that all patients experienced fatigue, with the other prevalent adverse events being anorexia (83%), diarrhea (67%), and hypertension (33%). Significant hematologic toxicities and severe fatigue, as well as cardiac arrhythmias, were observed, although the rate of treatment discontinuation was not specified.

Sherman et al. [22] reported high rates of grade 3–5 adverse events in both the pazopanib and placebo arms, affecting 88.9% and 85.3% of patients, respectively, with dysphagia, radiation dermatitis, liver enzyme elevations, and oral mucositis being common. One patient in the pazopanib arm suffered a grade 5 event related to sepsis. De la Fouchardière et al. [23] saw hypertension (50%), diarrhea (86%), and AST/ALT increase (44%) as common side effects, with 54% of patients experiencing grade 3–4 adverse events before randomization. The discontinuation rates were 38% in the intermittent pazopanib (IP) and 34% in the continuous pazopanib (CP) arms.

Bible et al. [24] reported frequent occurrences of hypertension (53%), fatigue (73%), and diarrhea (47%). Grade 3–4 hypertension and pharyngolaryngeal pain were noted in 13% of patients, and there was one death related to tumor hemorrhage. Some patients discontinued treatment due to adverse events, although the exact percentage is not specified. Lastly, Bible et al. [25] documented hypertension (71.7%), fatigue (78.3%), and diarrhea (75%) as common adverse effects, with significant events like grade 3–5 hypertension in 21.7% and fatigue in 8.3% of patients. Two deaths were possibly related to treatment, and 10.3% of patients discontinued due to adverse events.

## 4. Discussion

### 4.1. Summary of Evidence

This systematic review evaluated the efficacy and safety of pazopanib in thyroid cancer across six studies involving 445 patients. The findings indicate that pazopanib demonstrates clinical activity in RAI-refractory DTC, with the partial response rates ranging from 35.6% to 49% and with a median PFS period of around 11 months. In metastatic MTC, pazopanib showed a modest partial response rate of 14.3%, with median PFS and OS periods of 9.4 and 19.9 months, respectively. However, pazopanib showed minimal efficacy in ATC, with no confirmed RECIST responses and a median PFS period of only 2.1 months.

In RAI-refractory DTC, pazopanib’s efficacy is comparable to other TKIs such as sorafenib and lenvatinib, which are FDA-approved for this indication. The studies by Bible et al. [11,25] confirmed the clinical activity of pazopanib, even in patients who had received prior systemic therapies. The adverse events associated with pazopanib treatment highlight significant dose-dependent variability and the challenge of managing treatment toxicity. The common adverse events reported in studies, such as hypertension, diarrhea, and fatigue, frequently occur across various dosages. For instance, in the study by Bible et al. [11], hypertension was noted in 47% of patients, while in the study by Bible et al. [25], it was reported in as many as 71.7% of participants. Similarly, fatigue was universally high in the study by Chow et al. [21], affecting 100% of patients. The severe adverse events (Grade 3–5) varied considerably, with Sherman et al. [22] reporting a high incidence rate of 88.9% in the pazopanib arm. The treatment discontinuation rates due to adverse events also differed, with up to 14% of patients discontinuing treatment in the Bible et al. [11] study, reflecting the balance required between managing efficacy and minimizing toxicity to maintain patient quality of life.

In MTC patients, pazopanib demonstrated modest efficacy. Bible et al. [23] reported a partial response rate of 14.3%, indicating potential clinical activity, but further studies are needed to establish its role in MTC treatment. The safety profile was acceptable, with manageable toxicities. Moreover, in ATC, pazopanib showed minimal single-agent activity. Bible et al. [24] reported no confirmed RECIST responses and a median survival time of 111 days. Given the aggressive nature of ATC and poor prognosis, these findings suggest that pazopanib monotherapy is not effective in this setting.

The PAZOTHYR study by de la Fouchardière et al. [23] investigated the intermittent versus continuous dosing of pazopanib in RAI-refractory DTC. The results showed no significant difference in time to treatment failure, PFS, or overall response rate between the two dosing schedules. Intermittent dosing did not lead to improved tolerability or efficacy, indicating that continuous dosing remains the preferred approach.

The studies also explored potential predictive biomarkers for response to pazopanib. Bible et al. [25] assessed early changes in thyroglobulin (Tg) levels and mean corpuscular volume (MCV) as potential predictors of response but found no significant correlation. This highlights the need for further research to identify biomarkers that can predict which patients are most likely to benefit from pazopanib therapy.

In the investigation conducted by Ball et al. [26], the combination of trametinib, a MEK inhibitor, with pazopanib, a multikinase inhibitor, was explored due to their respective influences on the RAS/RAF/MEK/ERK signaling pathway. Trametinib alone showed a potent inhibitory effect with growth inhibition 50 (GI50) rates ranging between 1.1 and 4.8 nM, while pazopanib exhibited a moderate effect, with the GI50 rates ranging from 1.4 to 7.1 µM. The combination therapy notably led to a sustained reduction in tumor volume by over 50% in xenograft models bearing KRASG12R or BRAFV600E mutations, which underscores the effectiveness of targeting multiple pathways in thyroid cancer.

In a similar manner, the study by Di Desidero et al. [27] examined the impact of pazopanib in combination with topotecan, a topoisomerase inhibitor, on anaplastic thyroid cancer cells. Their findings demonstrated a strong synergism between the drugs, significantly reducing cell proliferation and decreasing the expression of critical genes such as ABCG-2, VEGF, HIF-1α, and CSF-1. The observed synergistic effect suggests that the combination of pazopanib and topotecan could be a promising therapeutic strategy for ATC, a particularly aggressive and treatment-resistant form of thyroid cancer.

Relevant to this review, the studies by Milling et al. [28] and Ferrari et al. [29] provided significant insights into the treatment of advanced thyroid cancers with tyrosine kinase inhibitors (TKIs). Milling et al. [28] focused on the efficacy of pazopanib, cabozantinib, and vandetanib in treating medullary thyroid cancer, highlighting their potential for increased progression-free survival and overall survival. The study also discussed the common adverse effects associated with these TKIs, particularly hypertension, which complicates treatment due to its contribution to cardiovascular disease. This side effect remains a considerable challenge as its mechanisms are poorly understood, and the management approach is generally symptomatic.

In a similar manner, the study by Ferrari et al. [29] investigated the effects of pazopanib on primary human anaplastic thyroid cancer cells in vitro, marking the first report of pazopanib’s antineoplastic activities in primary ATC cells. The study demonstrated pazopanib’s ability to inhibit cell proliferation, migration, and invasion and to induce apoptosis, alongside a significant reduction in VEGF expression. These results underscore pazopanib’s potential as a valuable treatment option for ATC, a form of thyroid cancer known for its aggressiveness and limited treatment success.

The failure of synergism between pazopanib and other drugs can be attributed to several factors including pharmacokinetic interactions, where drugs influence the metabolism of pazopanib, leading to altered efficacy or toxicity. Additionally, overlapping toxicity profiles exacerbate side effects, limiting the tolerable doses. Mechanistic antagonism may also occur, where the actions of combined drugs counteract each other, undermining their therapeutic effect. Complex disease pathways and individual patient variability further complicate the effective integration of pazopanib with other treatments, often resulting in unpredictable outcomes.

Nevertheless, the failure of chemotherapy in treating thyroid cancer is largely attributed to the unique biological characteristics of thyroid cells and the nature of the cancer itself. Thyroid cancers, particularly differentiated types such as papillary and follicular thyroid cancers, are often radioiodine-responsive and do not respond well to conventional chemotherapy [30,31]. These cells exhibit a low mitotic index, which reduces the effectiveness of chemotherapy drugs that target rapidly dividing cells. Additionally, chemotherapy resistance can arise from mutations in tumor suppressor genes and alterations in drug metabolism pathways within the cancer cells [32]. Different treatment regimens have proved to be more or less efficient. However, for papillary thyroid cancer, the most common type, the five-year survival rate is over 99% for localized disease [33]. Follicular thyroid cancer also has high survival rates, nearing 98% for localized cases [33]. Medullary thyroid cancer’s five-year survival rate is around 86% for localized disease but decreases with progression. Anaplastic thyroid cancer, although rare, is highly aggressive, with a five-year survival rate below 20%, reflecting its poor prognosis [34].

The study outcomes from the systematic review demonstrate the variability in pazopanib’s efficacy and tolerability across different settings and patient cohorts in thyroid cancer treatment. The studies by Bible et al. [11,25], showing a significant partial response rates and relatively longer median progression-free survival (PFS), suggest that pazopanib has a potential benefit in RAI-refractory differentiated thyroid carcinoma (DTC) when patients can tolerate the treatment. However, the minimal impact on iodine uptake observed in the study by Chow et al. [21] and the limited efficacy in aggressive cancers such as anaplastic thyroid carcinoma (ATC), as reported by Sherman et al. [22], highlight the drug’s limitations in altering fundamental disease processes or in more resistant cancer forms. Further, the varying outcomes also reflect the impact of pazopanib’s safety profile, where significant adverse events such as hypertension, diarrhea, and fatigue, as well as serious events leading to treatment discontinuation, challenge its long-term use. The data underline the need for careful patient selection and management strategies to optimize pazopanib’s therapeutic potential while minimizing its adverse effects.

Pazopanib’s selectivity towards thyroid cancer stems from its ability to inhibit key tyrosine kinase pathways, such as VEGFR, PDGFR, and c-Kit, which are crucial in thyroid cancer pathogenesis due to frequent mutations and overexpression in these pathways. Thyroid cancer, particularly given its high vascular nature, relies heavily on angiogenesis for growth and metastasis, making it particularly susceptible to pazopanib’s anti-angiogenic effects. This contrasts with other cancers that may not have the same dependence on vascular pathways or exhibit different mutational profiles, rendering pazopanib less effective outside of the thyroid cancer context.

### 4.2. Limitations and Future Directions

Several limitations should be considered when interpreting the results of this systematic review. First, the included studies varied in their designs, patient populations, and endpoints, which may have introduced heterogeneity. The sample sizes in some studies were small, particularly in the phase I trial by Chow et al. [21] and the ATC study by Bible et al. [24], limiting the generalizability of the findings. Second, there were a lack of randomized controlled trials comparing pazopanib directly with other standard treatments, which made it challenging to determine its relative efficacy. Additionally, potential publication bias cannot be excluded, as studies with negative results may be less likely to be published.

The future research on pazopanib in thyroid cancer should focus on optimizing the dosing schedules and combinations with other therapeutics for enhanced efficacy and reduced toxicity. Identifying biomarkers for response and resistance could also guide patient selection and personalized treatment strategies. Additionally, exploring the molecular mechanisms of pazopanib resistance might provide insights into novel targets and improve outcomes in refractory cases.

## 5. Conclusions

Pazopanib shows unclear efficacy and clinical activity in patients with RAI-refractory differentiated thyroid carcinoma, with manageable toxicity profiles. It offers a potential therapeutic option for patients who have progressed on or are intolerant to other TKIs. In medullary thyroid carcinoma, pazopanib demonstrates modest efficacy but further studies are needed to confirm its role. Pazopanib has minimal efficacy in anaplastic thyroid carcinoma and is not recommended as a monotherapy in this setting. The intermittent dosing of pazopanib does not improve the outcomes compared to continuous dosing. Further large-scale randomized trials are warranted to establish the optimal use of pazopanib in thyroid cancer treatment and to explore predictive biomarkers for responses.

## Figures and Tables

**Table 1:** Characteristics of the included studies.

Country	Year	Study Design	Sample Size	Study Quality	Study and Author
USA	2010	Multicenter Phase II Trial	37	High	Bible et al. [11]
USA	2017	Phase I Trial	6	Moderate	Chow et al. [21]
USA	2023	Randomized Phase II Trial	71	High	Sherman et al. [22]
France	2021	Randomized Phase II Trial (PAZOTHYR)	100	High	de la Fouchardière et al. [23]
USA	2012	Multicenter Phase II Trial	15	High	Bible et al. [24]
Multi-country	2020	International Phase II Trial	60	High	Bible et al. [25]

**Table 2:** Patient demographics and baseline characteristics.

Sample Size	Mean Age (Years)	Gender (Male/Female)	Thyroid Cancer Type	Prior Treatments	Study and Author
37	Median 59 (range 25–82)	17/20	RAI-refractory DTC	Surgery, RAI, some prior MKIs	Bible et al. [11]
6	Mean 57.5 (range 37–72)	4/2	Well-differentiated thyroid carcinoma (WDTC)	Surgery, RAI, some prior TKIs	Chow et al. [21]
89 (71 eligible)	Median 65 (IQR 58–68)	34/37	Anaplastic thyroid carcinoma (ATC)	Surgery, no prior systemic therapy for ATC	Sherman et al. [22]
168 (100 randomized)	Median 67 (range 34–85)	81/87	RAI-refractory DTC	Surgery, RAI, some prior MKIs	de la Fouchardière et al. [23]
15	Median 66 (range 45–77)	5/10	Anaplastic thyroid carcinoma (ATC)	Surgery, radiation, prior systemic therapies	Bible et al. [24]
60	Median 60 (25th–75th percentile 51–69)	33/27	RAI-refractory DTC	Surgery, RAI, prior systemic therapies	Bible et al. [25]

DTC—differentiated thyroid carcinoma; WDTC—well-differentiated thyroid carcinoma; ATC—anaplastic thyroid carcinoma; RAI—radioactive iodine; MKIs—molecular kinase inhibitors; TKIs—tyrosine kinase inhibitors; IQR—interquartile range.

**Table 3:** Treatment details and efficacy outcomes.

Study and Author	Pazopanib Dosage	Treatment Duration	Efficacy Outcomes
Bible et al. [11]	800 mg daily until progression or intolerability	Median PFS 11.7 months	Partial response rate of 49%; median OS not reached
Chow et al. [21]	800 mg daily for 12 weeks	Median PFS 6.7 months	4/5 (80%) achieved stable disease; no significant impact on iodine uptake
Sherman et al. [22]	400 mg daily pre-IMRT, 300 mg during IMRT	Pazopanib arm median OS 5.7 months	No significant difference in OS between pazopanib and placebo arms
de la Fouchardière et al. [23]	800 mg daily (continuous or intermittent)	Median TTF not significantly different	ORR 5% post-randomization; intermittent dosing not superior
Bible et al. [24]	800 mg daily until progression or intolerability	Median PFS 2.1 months	No confirmed RECIST responses; minimal clinical activity
Bible et al. [25]	800 mg daily until progression or intolerability	Median PFS 11.4 months	Partial response rate of 36.7%; median OS 2.6 years

PFS—progression-free survival; OS—overall survival; IMRT—intensity-modulated radiation therapy; TTF—time to treatment failure; ORR—overall response rate; RECIST—Response Evaluation Criteria in Solid Tumors.

**Table 4:** Safety profiles and adverse events.

Study and Author	Common Adverse Events	Grade 3–5 Adverse Events	Treatment Discontinuation Due to AEs
Bible et al. [11]	Hypertension (47%), Diarrhea (49%), Fatigue (51%)	Grade 3–4 AEs in 46%; one grade 5 AE (pulmonary hemorrhage)	14% discontinued due to AEs
Chow et al. [21]	Fatigue (100%), Anorexia (83%), Diarrhea (67%), Hypertension (33%)	Grade 3–4 hematologic toxicity, fatigue, arrhythmia	Not specified
Sherman et al. [22]	Dysphagia, Radiation Dermatitis, ALT/AST Elevation, Oral Mucositis	Grade 3–5 AEs in 88.9% (pazopanib), 85.3% (placebo); one grade 5 AE (sepsis) in pazopanib arm	Not specified
de la Fouchardière et al. [23]	Hypertension (50%), Diarrhea (86%), AST/ALT Increase (44%)	Grade 3–4 AEs in 54% (before randomization); no significant difference between arms	38% (IP), 34% (CP) experienced grade 3–4 AEs
Bible et al. [24]	Hypertension (53%), Fatigue (73%), Diarrhea (47%)	Grade 3–4 hypertension (13%), pharyngolaryngeal pain (13%); one death due to tumor hemorrhage	Treatment discontinued in some due to AEs
Bible et al. [25]	Hypertension (71.7%), Fatigue (78.3%), Diarrhea (75%)	Grade 3–5 hypertension (21.7%), fatigue (8.3%); two deaths possibly related	10.3% discontinued due to AEs

AEs—adverse events; ALT—alanine aminotransferase; AST—aspartate aminotransferase; IP—intermittent pazopanib; CP—continuous pazopanib.
